# A herbivore-induced plant volatile interferes with host plant and mate location in moths through suppression of olfactory signalling pathways

**DOI:** 10.1186/s12915-015-0188-3

**Published:** 2015-09-16

**Authors:** Eduardo Hatano, Ahmed M. Saveer, Felipe Borrero-Echeverry, Martin Strauch, Ali Zakir, Marie Bengtsson, Rickard Ignell, Peter Anderson, Paul G. Becher, Peter Witzgall, Teun Dekker

**Affiliations:** Department of Plant Protection Biology, Swedish University of Agricultural Sciences, Box 102, 23053 Alnarp, Sweden; Present address: Department of Biological Sciences, Vanderbilt University, Nashville, TN 37235 USA; Biological Control Laboratory, Colombian Corporation for Agricultural Research, Km 14 via Mosquera-Bogotá, Mosquera, Colombia; Fachbereich Biologie, Universität Konstanz, 78457 Konstanz, Germany; Present address: Institute of Imaging & Computer Vision, RWTH Aachen University, Kopernikusstr. 16, 52074 Aachen, Germany; Present address: Department of Environmental Sciences, COMSATS Institute of Information Technology, Vehari, Pakistan

**Keywords:** (*E*)-4,8-dimethyl-1,3,7-nonatriene, Herbivore-induced plant volatiles, Suppression, Antennal lobe, *Spodoptera littoralis*, Oviposition choice, Mating disruption, Olfaction, Orientation

## Abstract

**Background:**

Plants under herbivore attack release volatiles that attract natural enemies, and herbivores in turn avoid such plants. Whilst herbivore-induced plant volatile blends appeared to reduce the attractiveness of host plants to herbivores, the volatiles that are key in this process and particularly the way in which deterrence is coded in the olfactory system are largely unknown. Here we demonstrate that herbivore-induced cotton volatiles suppress orientation of the moth *Spodoptera littoralis* to host plants and mates.

**Results:**

We found that (*E*)-4,8-dimethyl-1,3,7-nonatriene (DMNT), an induced volatile, is key in herbivore deterrence: DMNT suppressed plant odour- and pheromone-induced behaviours. We then dissected the neurophysiological basis of this interaction. DMNT-responding glomeruli were also activated by other plant compounds, suggesting that *S. littoralis* possesses no segregated olfactory circuit dedicated exclusively to DMNT. Instead, DMNT suppressed responses to the main pheromone component, (*Z*)-9-(*E*)-11-tetradecenyl acetate, and primarily to (*Z*)-3-hexenyl acetate, a host plant attractant.

**Conclusion:**

Our study shows that olfactory sensory inhibition, which has previously been reported without reference to an animal’s ecology, can be at the core of coding of ecologically relevant odours. As DMNT attracts natural enemies and deters herbivores, it may be useful in the development or enhancement of push-pull strategies for sustainable agriculture.

**Electronic supplementary material:**

The online version of this article (doi:10.1186/s12915-015-0188-3) contains supplementary material, which is available to authorized users.

## Background

Choosing suitable oviposition sites is a fundamental strategy and for most species the only care provided for their offspring [1]. Therefore, animals should carefully assess cues that signify the quality of the site, the risk of predation, and the likelihood of competition. Although experience may refine an animal’s responses [2], these signals often trigger innate responses through hard-wired neural circuitries [3].

Plants emit a plethora of volatile organic compounds (VOCs) that herbivorous insects use as orientation cues [4]. Herbivore damage induces plants to activate specific biochemical pathways that heighten their defence against herbivores. In addition to toxins and antifeedants, induced defences can also be indirect by recruiting natural enemies of herbivores using herbivore-induced plant volatiles (HIPVs). These are generally composed of green leaf volatiles (C6 molecules) and a set of terpenoids [5–7]. Parasitoids and predators cue in to several of these compounds [5, 8], of which the most well studied is the *de novo* synthesized homoterpene, (*E*)-4,8-dimethyl-1,3,7-nonatriene (DMNT) [5, 9].

Herbivores themselves are also sensitive to HIPVs, and prefer plants that do not emit these odours [10, 11]. Detection and avoidance of induced plants has benefits for ovipositing herbivores. (I) HIPVs indicate intra- or interspecific competitors in the host plant site [12, 13]. (II) They indicate a heightened defence response in attacked plants [14, 15] and frequently in its downwind neighbours [16, 17], which generally affects survival, particularly of early larval instars [18]. (III) Finally, offspring in sites containing induced plants will likely suffer a higher level of parasitization and predation [11, 19].

Several studies have demonstrated the significance of odours in aversion of induced plants [12, 13, 20]. Yet, herbivore detection of HIPVs has not been dissected out either behaviourally or physiologically, in spite of their fundamental role in push-pull systems and their potential for novel chemical ecology-based methods of insect control [21].

In this study we show that DMNT, a key plant compound used by natural enemies in finding prey, is used by females and males of the Egyptian cotton leaf worm (*Spodoptera littoralis*, Boisd., Lepidoptera: Noctuidae), to avoid induced plant sites and calling females, respectively. Finally, we found that behavioural disruption is paralleled by suppression of pheromone and plant odour induced-activity in the antennal lobes (ALs) and antennae without any DMNT-specific neural channel. Olfactory sensory inhibition appears of behavioural and evolutionary ecological significance, and might benefit fitness of herbivores by assessing risks of predation and competition. The importance of the findings is also discussed in the light of sustainable agriculture.

## Results

### Herbivore-damaged plants affect behaviour

**Fig. 1:**
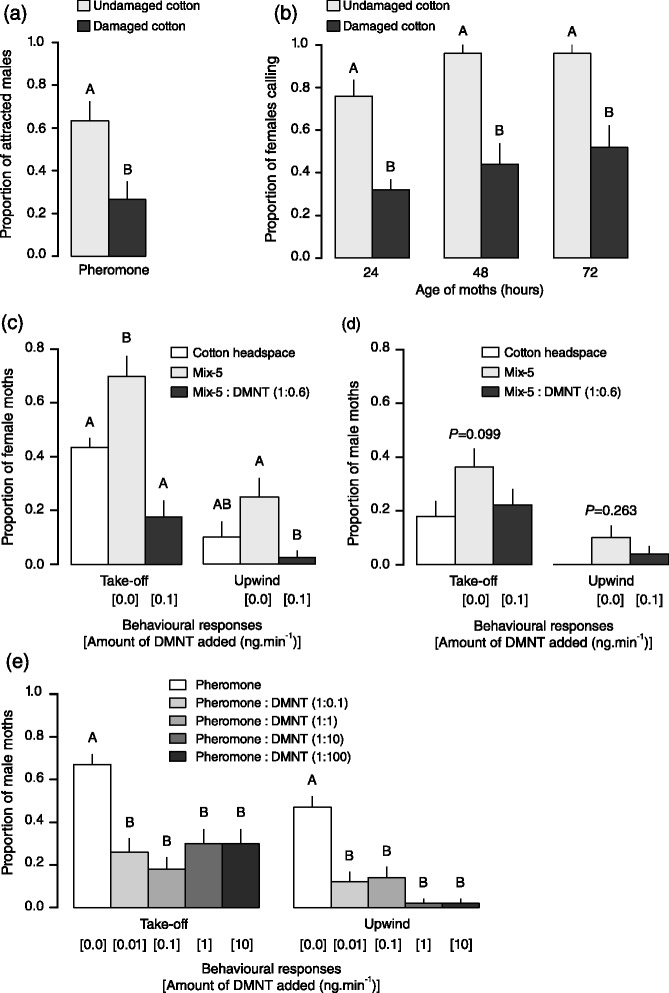
Behavioural responses of male and female *S. littoralis* to odours from plants or synthetic blends. Bars represent proportions of attracted moths (+SE). **a** Attraction of virgin male moths towards pheromone gland extracts in a wind tunnel assay with herbivore-damaged or undamaged cotton plants as odour background (*P <* 0.01, n = 30). **b** Proportion of virgin female moths that exhibited calling behaviour in the presence of either damaged or undamaged cotton plants (n = 30). **c** Attraction of mated females to cotton headspace collection (data as in [30]), Mix-5 and Mix-5:DMNT (n = 40). **d** Attraction of virgin males to cotton headspace (data as in [30]), Mix-5 and Mix-5:DMNT were not significantly different (n = 50). **e** Pheromone-triggered response of males was suppressed by DMNT at different ratios (n = 50). Different letters indicate statistical differences between the odours (binomial GLM, *P* < 0.001)

According to previous studies, damaged and undamaged cotton plants emit different patterns of volatiles. A series of tests was performed to confirm that these patterns differentially affect the behavioural response of moths [22–28]. In wind tunnel dual-choice assays, a higher percentage of virgin males was attracted to the sex pheromone extract with odorant background from undamaged than from damaged plants (Fig. 1a; d.dev. = 8.352, df = 1, *P* < 0.01). When virgin females had access to either damaged or undamaged plants in cage assays, calling behaviour was suppressed when females perched on herbivore-induced plants compared to females on undamaged plants (Fig. 1b; d.dev. = 40.759, df = 1, *P* < 0.01). This effect was independent of female age (d.dev. = 2.021, df = 2, *P* = 0.364). These results confirmed that herbivore-induced cotton plants suppressed sexual behaviours in both males and females compared to undamaged plants. However, it remained to be tested which volatile compound suppressed these behavioural responses.

### DMNT effects on flight behaviour

We investigated the effect of the HIPV DMNT on the behaviour of male and female moths due to its emission in high amounts by damaged cotton plants [24–28] and its ecological importance in attracting natural enemies. Based on previous analyses of cotton VOCs [20, 29], we designed a synthetic plant blend (Mix-5) that optimized attraction of S*. littoralis* [20, 29, 30] and that could also be used in physiological assays. Mix-5 contained five volatile compounds found in the cotton headspace: β-myrcene, ocimene, (*R*)-(+)-limonene, (*Z*)-3-hexenyl acetate and nonanal.

In wind tunnel assays using a piezo-electric sprayer to disperse stimuli, cotton headspace collection (data as in [30]) triggered weaker behavioural responses of mated female moths than Mix-5 did (Fig. 1c). Addition of DMNT to Mix-5 strongly inhibited take-off and upwind flight steps (Fig. 1c; Take-off: d.dev. = 25.822, df = 2, *P* < 0.001; Upwind: d.dev. = 12.835, df = 2, *P* < 0.01).

Attraction of males to cotton headspace collection (data as in [30]) and Mix-5 was relatively weak compared to females, and it was not significantly affected by the addition of DMNT (Fig. 1d; Take-off: d.dev. = 4.618, df = 2, *P* = 0.099; Upwind: d.dev. = 7.272, df = 2, *P* = 0.263). Conversely, attraction to the main pheromone component, *Z*9,*E*11-14:OAc, was disrupted in the presence of DMNT at several concentrations (Fig. 1e; Take-off: d.dev. = 49.529, df = 4, *P* < 0.001; Upwind: d.dev. = 70.638, df = 4, *P* < 0.001).

These findings demonstrate that DMNT is an active compound in the cotton volatile blend that significantly interferes in odorant perception in both sexes, affecting their mating and oviposition choices. Next we investigated how DMNT affected odour coding in males and females.

### DMNT suppressed odour-evoked Ca^2+^ responses in males

**Fig. 2:**
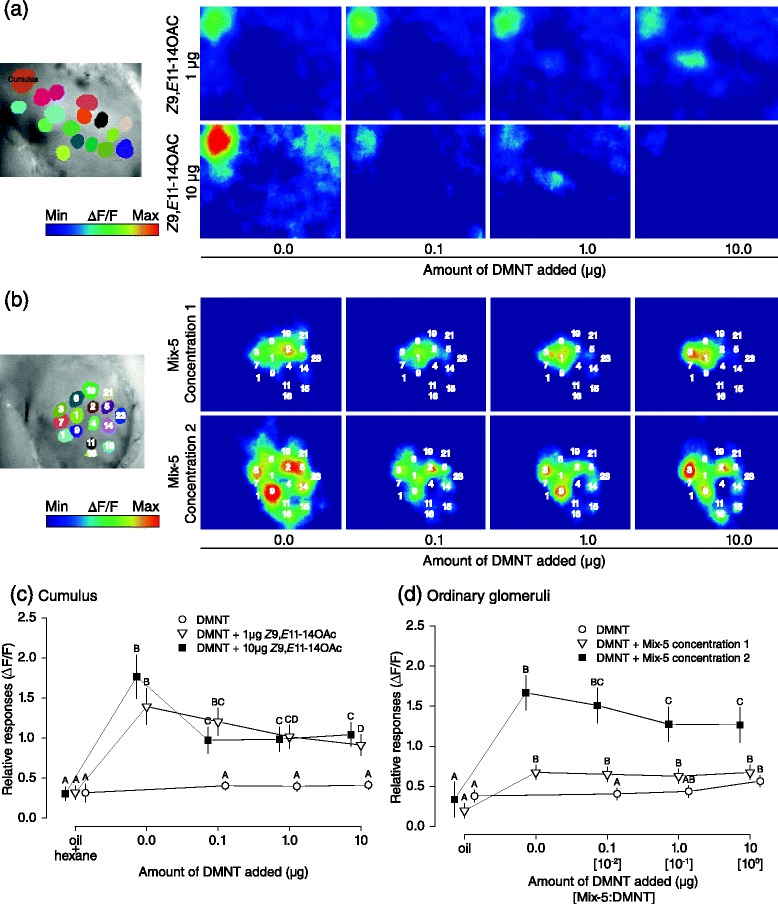
Effect of DMNT on Ca^2+^ responses in male AL to *Z*9,*E*11-14:OAc and Mix-5. Images were collectively scaled to strongest activation, and symbols represent mean values of maximum odour responses of identified glomeruli (+SE). **a** Computed map of glomeruli highlighting the area of the cumulus (*left*) and representative false colour-coded images of maximum Ca^2+^ responses to pheromone (1 and 10 μg) and DMNT (0.1–10 μg) (*right*). **b** Computed map showing the areas of ordinary glomeruli from which responses were calculated (*left*) and representative false colour-coded images of maximum Ca^2+^ responses to Mix-5 (concentrations 1 and 2) mixed with DMNT (0.1–10 μg) (*right*). **c** Ca^2+^ responses of the cumulus (n = 13) to 1 μg (open triangles), 10 μg pheromone (solid squares) and DMNT alone (open circles). **d** Ca^2+^ responses over all glomeruli (n = 76) from 13 moths to Mix-5 concentration 1 (open triangles), 2 (solid squares) and DMNT alone (open circles). Different letters denote significantly different responses (lme, *P <* 0.05) within treatments (groups of colour)

The largest glomerulus of the macroglomerular complex (MGC) of *S. littoralis*, the cumulus, is dedicated to detecting the main pheromone component, *Z*9,*E*11-14:OAc [31]. As the major pheromone alone induces robust upwind flight in males, we focussed on the responses of the cumulus to *Z*9,*E*11-14:OAc alone and in combination with DMNT using Ca^2+^ imaging. *Z*9,*E*11-14:OAc (1 and 10 μg) elicited calcium responses in the cumulus, but addition of DMNT suppressed them (Fig. 2a,c). Suppression was particularly strong at 10 μg of *Z*9,*E*11-14:OAc (linear mixed-effects models, lme, χ^2^ = 111.91, df = 4, *P* < 0.001) and more gradual at 1 μg with increasing doses of DMNT (lme, χ^2^ = 96.149, df = 4, *P* < 0.001). Response to DMNT alone was found only in ordinary glomeruli (Fig. 2d; lme, χ^2^ = 8.324, df = 3, *P* < 0.05), but not in the cumulus (Fig. 2c; lme, χ^2^ = 4.279, df = 3, *P* = 0.233). These results corroborate the inhibition of the olfactory-evoked behaviour of male moths when flying towards a pheromone source, and suggest that the pheromone and DMNT signals interact at the peripheral level, possibly either via competitive antagonism or allosteric inhibition, as was proposed for *Helitothis virescens* [32].

Male ordinary glomeruli responded to Mix-5 at both concentrations (Fig. 2b,d; lme, concentration 1: χ^2^ = 21.247, df = 4, *P* < 0.001; concentration 2: χ^2^ = 35.581, df = 4, *P* < 0.001). However, addition of DMNT to Mix-5 suppressed Ca^2+^ responses at Mix-5 only at concentration 2 but not at concentration 1 (Fig. 2b,d). Because of the weak behavioural responses to Mix-5 in males (Fig. 1d), we did not further investigate the activity of individual glomeruli.

### Suppression of pheromone perception occurs at the peripheral level

**Fig. 3:**
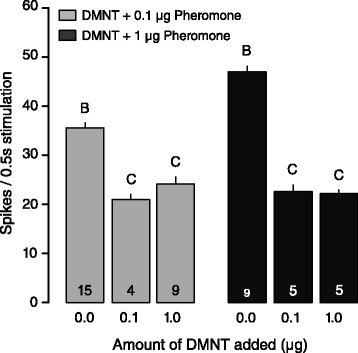
Responses of OSNs to the main pheromone component, *Z*9,*E*11-14:OAc, and DMNT in male *S. littoralis*. Response to control air was subtracted from stimulus responses. Bars represent the mean values (+SE) of spike frequency during stimulation. Different letters denote significantly different spike responses (lme, *P <* 0.05) within same treatments (groups of colour). N-values are indicated on bars

As responses from sensory neurons dominate Ca^2+^ responses in the ALs, we verified if the inhibition of the response in combination with DMNT was due to presynaptic (OSN-OSN or local interneuron-OSN) inhibition, or whether the interaction occurred peripherally. Recordings from male-specific long trichoid sensilla housing olfactory sensory neurons (OSNs) that responded solely to pheromone demonstrated that addition of DMNT to the pheromone stimulus significantly reduced the response to the latter at different concentrations (Fig. 3; Poisson glm, 0.1 μg: d.dev = 45.635, df = 2, *P* < 0.001; 1 μg: d.dev = 114.35, df = 2, *P* < 0.001). This result suggests that attenuation of the pheromone OSN firing response was independent of input from the DMNT OSN.

### DMNT suppressed odour-evoked Ca^2+^ responses in females

**Fig. 4:**
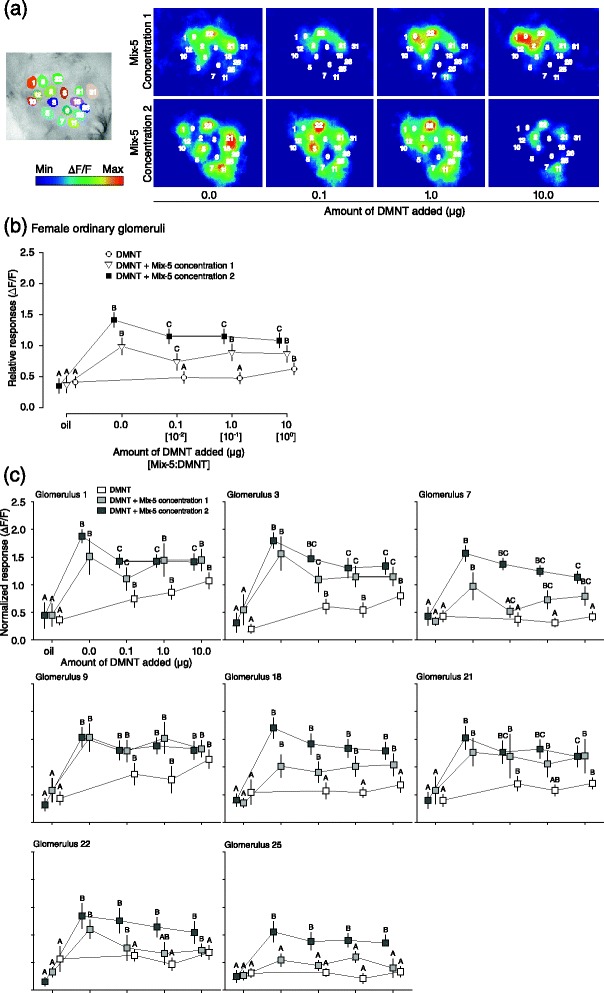
Effect of DMNT on Ca^2+^ responses in female ALs to the synthetic volatile mixtures. **a** Computed map showing the areas of ordinary glomeruli from which responses were calculated (*left*) and representative false colour-coded images of Ca^2+^ responses to Mix-5 (concentrations 1 and 2) and DMNT (0.1–10 μg) (*right*). Images were collectively scaled to strongest activation. **b** Ca^2+^ responses over all glomeruli (n = 81) from 13 moths to Mix-5 concentration 1 (open triangles), 2 (solid squares), mixed with DMNT (0.1 to 10 μg), and DMNT alone (open circles). Symbols represent the mean values of maximum Ca^2+^ response (+SE). **c** Ca^2+^ responses of individual ordinary glomeruli (n = 13) to Mix-5 concentration 1 (*light grey*), 2 (*dark grey*) mixed with DMNT, and DMNT alone (*white*). Squares represent the mean values of maximum relative Ca^2+^ response (+SE). Different letters denote significantly different responses (lme, *P <* 0.05) within treatments (groups of colour)

Ca^2+^ responses to both concentrations of Mix-5 in the ALs of females were suppressed by DMNT (Fig. 4; lme, concentration 1: χ^2^ = 32.199, df = 4, *P* < 0.001; concentration 2: χ^2^ = 53.514, df = 4, *P* < 0.001). DMNT triggered Ca^2+^ responses only at 10 μg, the highest dose (Fig. 4a,b; lme, χ^2^ = 8.832, df = 3, *P* < 0.001). It is likely that the expression of DMNT-responding ORs in males and females is quantitatively similar, since the olfactory system of males seems to be as sensitive to DMNT as females are in dose-responses to DMNT. Since attraction of *S. littoralis* females towards Mix-5 was strongly disrupted by DMNT (Fig. 1c), we investigated whether the suppression was due to a particular single glomerulus or set of glomeruli, and whether DMNT disrupted the perception of any particular odour in the mixture. All test statistics of responses of individual glomeruli are presented in Additional file 1.

Using a set of reference compounds, we mapped on average 16 responding glomeruli per animal. Based on activation patterns of reference compounds, eight glomeruli (glomeruli 1, 3, 7, 9, 18, 21, 22, and 25) were consistently identified. Glomeruli differed substantially in their response to Mix-5, DMNT and their combinations. Glomerulus 18 was not significantly suppressed by the addition of DMNT when responding to either concentration of Mix-5, unlike glomeruli 1, 3 and 25 (Fig. 4c). Glomeruli 7, 21 and 22 were intermediate between these two, with DMNT-induced suppression only at the lower dose of Mix-5 (Fig. 4c). Suppression was not restricted to those glomeruli that responded to DMNT (glomeruli 1, 3, 9, 21 and 22), indicating that besides competitive antagonism and allosteric interactions, other mechanisms may be involved in the suppressive activity of the DMNT-sensitive OSN, such as presynaptic inhibition via local interneuron (LNs) [33, 34].

**Fig. 5:**
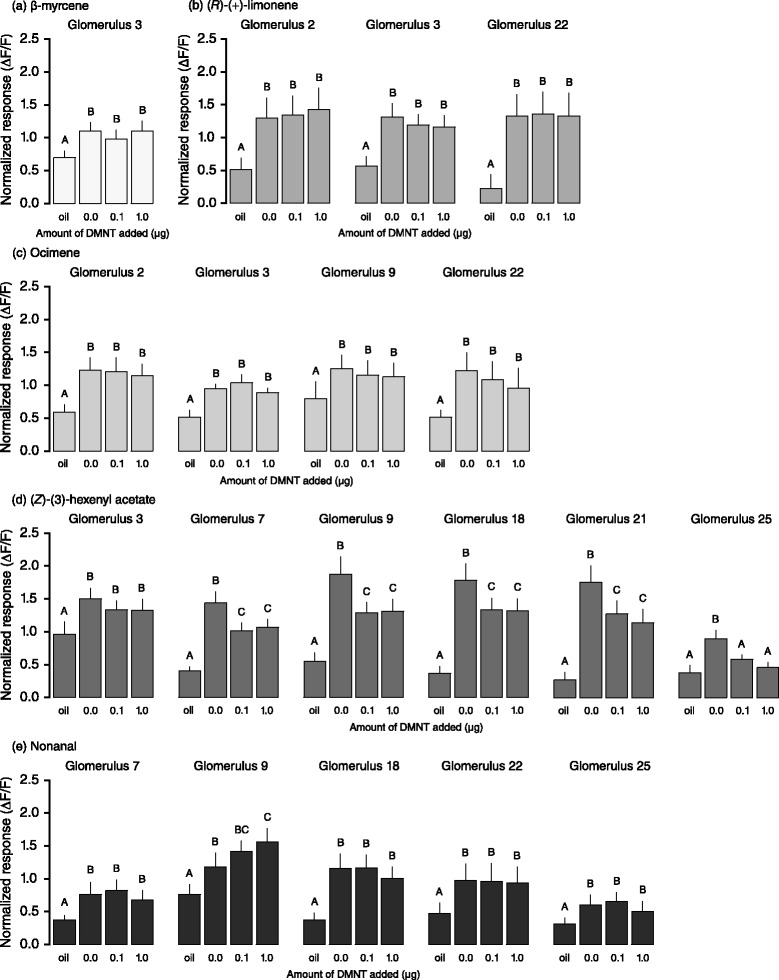
Ca^2+^ responses of female ordinary glomeruli to each single plant odorant (10 μg) mixed with DMNT (0.1–1.0 μg). **a.** β-myrcene, **b.** (*R*)-(+)-limonene, **c.** ocimene, **d. **(*Z*)-(3)-hexenyl acetate, **e. **nonanal. DMNT significantly suppressed the Ca^2+^ response to (Z)-3-hexenyl acetate responding glomeruli except for glomerulus 3. Responses to other odorants were not suppressed by DMNT. Bars represent the mean values of maximum relative Ca^2+^ response (+SE) of each identified glomeruli. Different letters denote significantly different calcium responses within odorants and glomeruli (lme, *P <* 0.05, n = 13)

Next, we investigated Ca^2+^ responses to single components in order to identify the response to which odorant of Mix-5 was affected by DMNT. Since significant suppression was already detected at 0.1 and 1.0 μg DMNT, we focussed on these two doses. Each compound from Mix-5 was tested at 10 μg, which elicited significant calcium responses (Fig. 5). Ca^2+^ responses showed that only the response to (*Z*)-3-hexenyl acetate was significantly suppressed by addition of DMNT (Fig. 5d) compared to the other compounds (Fig. 5a,b,c,e). Among six glomeruli activated by (*Z*)-3-hexenyl acetate, five were significantly suppressed by DMNT (glomeruli 7, 9, 18, 21 and 25; Fig. 5d). Again, this effect was not restricted to DMNT-activated glomeruli. Some apparent mixture interactions were also observed. For instance, DMNT-induced suppression of glomerulus 18 was only observed when stimulating with (*Z*)-3-hexenyl acetate, but not with Mix-5. The opposite was observed for glomerulus 3. It may thus be that the behavioural suppression by DMNT is not solely caused by suppression of responses in specific glomeruli, but is due to intricate ensemble effects.

### Effect of (*S*)-(+)-linalool and (*R*)-(−)-linalool on pheromone responses

**Fig. 6:**
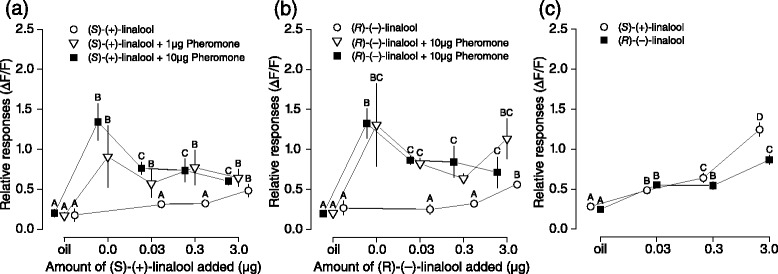
Effect of (*S*)-(+)-linalool and (*R*)-(−)-linalool on Ca^2+^ responses of male ALs to the pheromone component, (*Z*9,*E*11-14:OAc). Symbols represent the mean values of maximum relative Ca^2+^ response of identified glomeruli (+SE). **a** Maximum Ca^2+^ responses of the cumulus (n = 8) to pheromone (1 and 10 μg) mixed with (*S*)-(+)-linalool (0.03–3 μg). Response to increasing doses of (*R*)-(−)-linalool alone is shown with open circles. **b** Maximum Ca^2+^ responses of the cumulus to pheromone (1 and 10 μg) mixed with (*R*)-(−)-linalool (0.03 to 3 μg). Response to increasing doses of (*S*)-(+)-linalool alone is indicated by circles. **c** Ca^2+^ responses over all glomeruli (n = 93) from 8 moths to (*S*)-(+)-linalool (open circles) and (*R*)-(−)-linalool (solid squares). Different letters denote significantly different calcium responses (lme, *P <* 0.05) within the same treatments (groups of colour)

We compared the activity of DMNT to both linalool isomers, (*S*)-(+)-linalool and (*R*)-(−)-linalool, which were found to suppress pheromone-induced electrophysiological responses and oviposition [11, 32, 35, 36]. To compare responses, we compensated for the emission from oil of both linalool enantiomers compared to DMNT by reducing their concentration in solution. (*S*)-(+)-linalool and (*R*)-(−)-linalool suppressed pheromone-induced Ca^2+^ responses in male *S. littoralis* ALs. Both enantiomers suppressed Ca^2+^ responses from the cumulus to 10 μg pheromone (Fig. 6a,b; lme, (*S*)-(+)-linalool: χ^2^ = 50.532, df = 4, *P* < 0.001; (*R*)-(−)-linalool: χ^2^ = 51.942, df = 4, *P* < 0.001), in a similar way to DMNT (Fig. 2a,c). However, suppression at 1 μg pheromone was only observed with 0.3 μg of (*R*)-(−)-linalool (lme, χ^2^ = 28.302, df = 4, *P* < 0.001) and not with (*S*)-(−)-linalool (Fig. 6a,b; lme, χ^2^ = 19.611, df = 4, *P* < 0.001). Unlike DMNT, either isomer alone triggered a Ca^2+^ response in the cumulus (Fig. 6a,b; lme, (*S*)-(+)-linalool: χ^2^ = 12.297, df = 4, *P* < 0.01; (*R*)-(−)-linalool: χ^2^ = 12.82, df = 4, *P* < 0.01). Since pheromone-sensitive OSNs are finely tuned to pheromones only, it is possible that this phenomenon was caused by excessive stimulus fluxes.

In ordinary glomeruli, both linalool isomers triggered strong Ca^2+^ responses (Fig. 6c). Significant responses were observed from the lowest concentration up (lme, (*S*)-(+)-linalool: χ^2^ = 19.5, df = 3, *P* < 0.001; (*R*)-(−)-linalool: χ^2^ = 26.503, df = 3, *P* < 0.001), whereas DMNT triggered a response only at the highest concentration (Fig. 2d). Taken together, these results indicate that the olfactory system of *S. littoralis* is more sensitive to linalool than DMNT, whereas suppression by DMNT is stronger than by linalool. It is thus likely that DMNT is a more specific suppressive compound than both linalool isomers, and that the suppressive mechanism is highly sensitive to very low concentrations of DMNT.

## Discussion

Olfactory sensory systems constitute the neural interface between organisms and their odour environment. They selectively gate relevant ecological information and accordingly modulate behavioural responses [37]. Insects possess a multidimensional array of olfactory sensory neurons that registers fluxes and ratios of volatile compounds. The sensitivity of this array is thought to be finely tuned to the detection of ecologically relevant odours through a specific set of olfactory receptors they express [37]. This input is subsequently processed in the AL, the primary olfactory centre in the insect brain [37]. ALs in male moths are equipped with enlarged glomeruli called the macroglomerular complex (MGC), which receives exclusive input from pheromone-specific OSNs [38]. The remaining isomorphic glomeruli (63 glomeruli in *S. littoralis*; [31]) encode general odours such as plant VOCs.

HIPVs signal unfavourable environments and should, therefore, be detected and avoided. Although herbivores indeed appear to avoid HIPVs (this study; [12]), the specific compounds within the complex HIPV blend and the underlying neural mechanisms through which herbivore behaviour is suppressed were hitherto unknown. We demonstrate that DMNT is a key signal in the HIPV blend that induced suppression of host and mate orientation in *S. littoralis* to cotton and that this is mediated through neuronal inhibition of olfactory responses. We are currently investigating whether other cotton HIPVs, not included in the current study, may further augment behavioural inhibition towards induced cotton.

Cotton HIPVs redirect all basic odour-mediated behaviours in *S. littoralis*, that is, calling behaviour, oviposition, and orientation to cotton and partners (calling behaviour and pheromone attraction) (this study; [20, 39]). Using our cotton five-component mimic, we then demonstrated that DMNT, at ratios released by induced cotton, paralleled suppression of the above odour-mediated behaviours. Cotton systemically releases relatively large amounts of DMNT upon herbivore attack [20, 22, 27, 40], as do other plants (such as apple, maize, lima bean and cucumber) (review by [6]). More interestingly, natural enemies use DMNT to find their hosts [9, 41]. The evolutionary convergence of utilizing DMNT by both natural enemies and herbivores underlines its significance as a reliable signal in HIPV blends. HIPVs are diverse and consist of many compounds, some of which individually may not have a negative valence [42]. In contrast, DMNT appears highly characteristic for herbivore damage, released at the site of damage and systemically through induction of undamaged parts [25, 26], and even by neighbouring plants after volatile priming [16].

We addressed how the moth encodes DMNT. The reigning paradigm is that ecologically relevant odours are either coded through an activation pattern across different sensory neuron types (combinatorial coding) or through narrowly tuned single-odour-single-neuron systems (labeled-line coding) [37]. In *Drosophila*, highly relevant negative stimuli are coded via activation of single receptor neuron types, and directly mediate repulsion [43]. However, DMNT detection in *S. littoralis* appears not to be coded via a dedicated repulsion-inducing sensory neuron type. Instead, our recordings appear to support that DMNT acts through suppression of the activity of receptor neurons tuned to attractive compounds. This is to our knowledge the first demonstration that suppression of OSN activity is of fundamental behavioural, ecological and evolutionary significance. Furthermore, it places decades-old records of suppression of pheromone neuron activity by linalool and related compounds in an ecological framework, that is, tritrophic interactions. These earlier studies were conducted from a neurophysiological rather than an ecological perspective [36, 44–46]. The strong behavioural and ecological effects may in fact well be the evolutionary raison-d’-être of these interactions in the first place. Although other insect species may have evolved sensory channels dedicated to DMNT detection, our results demonstrate that detection of ecologically highly relevant odours does not require these channels, but instead can be encoded via combinatorial suppression of odour-evoked responses. In an ecological context, attenuating odour-evoked responses and attraction may prevent full HIPV-induced shutdown of host plant and mate orientation. This could be evolutionarily advantageous at high herbivore densities, and prevent herbivores from reproducing at all in the continuous presence of HIPVs.

Odour interactions can occur at various levels in the olfactory circuitry [38, 47]. Suppression of pheromone- and plant odour-evoked responses by DMNT appears to be a peripheral event, as evidenced by our single sensillum and Ca^2+^ imaging recordings. Suppression of OSN input has been a frequently observed phenomenon in arthropods [38, 48, 49] and mammals [50, 51]. In insects, suppression of pheromone antennal responses has long been documented [52], and ever since several plant and microorganism odorants have been reported as suppressors of OSN responses, for example, geraniol, linalool and β-ocimene. However, the majority of studies focussed on either behavioural responses or physiological mechanism of olfactory suppression, and few combined different approaches to finely elucidate the effect of olfactory coding on orientation and ecology [53, 54].

Some possible mechanisms for response suppression include competitive antagonism [32, 55, 56], allosteric inhibition [32, 55], presynaptic inhibition [33, 34, 57, 58] or ephaptic interactions [59]. The latter mechanism was demonstrated in *D. melanogaster*, in which the response of OSNs may inhibit the activity of sensilla co-located independently of synapses [59]. In our experiment using binary mixtures of plant odorants, (*Z*)-3-hexenyl acetate was the only component whose activity was significantly suppressed by DMNT. However, no sensillum type in *S. littoralis* was found responding to both compounds [60]. It is thus unlikely that DMNT suppresses odorant perception through ephaptic interaction. Therefore, the mechanism behind DMNT-mediated OSN suppression is likely competitive antagonism, allosteric inhibition [32] or presynaptic inhibition [33]. The mode of interaction between DMNT and pheromone or plant odours is as of yet uncertain, but this interaction may also be peripheral and mediated through similar processes. To answer this question, a large deorphanization project on *S. littoralis* receptors will shed further light on this in the near future (W.B. Walker, personal communication). It is important to note here that plant species differ in the precise ratio of terpenoids released as part of the HIPV blend [4]. We anticipate that other terpenoids can substitute for the ecological role of DMNT, depending on the plant species.

The use of odours in ‘jamming’ olfactory perception has in recent years been explored in the context of combatting pest insects. A search for an olfactory receptor co-receptor (ORCO) agonist and antagonist yielded several potential candidates for use in olfactory interference [61]. Similarly, several agonist and antagonist receptors for carbon dioxide, a host-signifying compound for blood-seeking insects [62], were successfully used in lowering biting incidence of mosquitoes under semi-field conditions [63, 64]. Here we demonstrate that jamming of olfactory perception also occurs naturally, as a part of olfactory coding of complex natural blends. In fact, it may well be a key component in the associational resistance in the push-pull cropping system in Kenya [65]. Intercropping of maize with *Melinis minutiflora* [66], intercrops that constitutively release DMNT, reduce maize borer *Chilo partellus* infestations [65]. This may well be caused by DMNT-induced suppression of host plant attraction and sexual communication in *C. partellus*. This parallel seems warranted, given the fact that olfactory coding in related moths is highly similar [67]. DMNT is also critical in attraction of *C. partellus* parasitoids [9]. This suggests that in the maize intercropping system DMNT fulfils both a push and a pull function.

## Conclusions

A single HIPV, DMNT, affects olfactory-triggered orientation of carnivorous and herbivorous insects of both sexes and, therefore, has a central role in shaping tritrophic interactions. To disrupt olfactory coding, a specialized dedicated channel is not necessary to suppress signalling input of ecologically relevant odorants. Further experiments should investigate whether this DMNT suppressive mechanism is conserved in other herbivore species. In addition, future studies should explore the potential application of DMNT, and possibly other HIPVs that induce odour-based associational resistance, to improve push-pull methodology and support sustainable food production.

## Methods

### Plants

Cotton seedlings (*Gossypium hirsutum* L., cv. Delta Pineland 90) were grown singly at 25 ± 5 **°**C and at 70 ± 5 % RH, under daylight and artificial light (400 W). Cotton plants used in behavioural experiments had eight to ten fully developed true leaves.

To produce damaged plants, two to three second to third instar larvae of *S. littoralis* were released on the second true leaf of the plant 24 h prior to experiments. Leaves and larvae were then enclosed inside fine mesh (0.2 × 0.2 mm) bags.

### Insects

*S. littoralis* eggs were obtained from cotton fields in El-Shatby and the lab culture at Assiut University, Egypt. Larvae were fed on a standard noctuid artificial agar-based diet, under a 16 L:8D photoperiod, at 24 **°**C. Males and females were separated as pupae into plastic boxes (30 × 20 × 10 cm) to obtain virgin insects and were kept in separate rooms in order to avoid pre-exposing males to pheromone before experiments. Virgin males and 24- to 27-h post-mated female moths were used in all bioassays when they were 2 to 3 days old. Adults had access to water *ad libitum*.

### Cage bioassay

Individual damaged or undamaged cotton plants were placed inside Plexiglas® cages (40 × 40 × 80 cm) and maintained in a ventilated climate chamber at 25 ± 2 **°**C, 65 ± 5 % RH, with a photoperiod of 16 L:8D. These cages were used to confine plant volatiles and to prevent interference between replicates.

Groups of five moths were transferred to cages at the start of the scotophase and observed during the following scotophase at 15-min intervals. Numbers of female moths exposing their abdominal pheromone gland were recorded at each observation. Moths and plants were replaced by new ones in all cages constituting a new replicate. Females of three different ages (24, 48, and 72 h old) were observed (N = 30).

### Wind tunnel bioassay

Adults were transferred individually to 2.5 × 12.5 cm glass tubes closed with gauze. Moths were kept in the wind tunnel room for at least 1 h before testing to acclimatize them to the testing environment. Experiments were carried out between 1 and 4 h after the onset of the scotophase.

Bioassays were performed in a Plexiglas wind tunnel (180 × 90 × 60 cm) with illumination at 6 lux, with a wind speed of 30 cm.s^−1^, at 24 ± 2 **°**C and 60 ± 10 % RH. Incoming air was filtered with active charcoal and outgoing air was extracted at an equal rate passing through a similar filtering system.

Glass tubes, with the gauze removed, were placed on a platform at the downwind end of the tunnel, and moths were observed for 5 min. Moths that showed oriented upwind flight up to half of the wind tunnel towards the odour source were counted as responding individuals. Three different bioassays were conducted: (I) attraction of males to pheromone gland extracts in the presence of damaged and undamaged cotton plants, (II) attraction to synthetic mixtures (plant components and pheromone) and cotton headspace collection of virgin males and (III) mated females.

#### Pheromones and plants

Pheromone gland extracts were prepared from 20 female glands collected 3 h into the scotophase and immersed in 20 μL redistilled hexane for 10 min. Three different extracts were pooled. The combined gland extract was diluted with hexane to a concentration of 2 ng.μL^−1^ based on the main component according to GC-MS analyses, and one female equivalent dose was established as 10 μL of the gland extract. All extracts and dilutions were stored at −18 **°**C.

In the wind tunnel, one undamaged and one damaged plant were placed 15 cm apart from each other. Fifteen centimetres downwind from each plant, a piece of filter paper (0.5 × 1.0 cm) loaded with 1 female gland equivalent dose was placed 30 cm above the wind tunnel floor. New filter papers with fresh pheromone gland extracts were introduced every 12 min, and the position of plants was reversed every five replications.

#### Synthetic mixtures and plant odours

Cotton odour was collected using a setup for headspace collection [29]. Volatiles were entrapped in glass filters containing 50 mg Super Q adsorbent (80/100 mesh, Altech, Deerfield, IL, USA) under 12 L:12D photoperiod, for 24 h, at 22 °C from individual plants. Headspace was collected during a total of 1,848 h from 40 plants. Filters were eluted with 500 mL re-distilled n-hexane and condensed under a stream of nitrogen. Samples were stored at −18 **°**C.

Eleven antennal active volatile components from damaged and undamaged cotton plants were previously identified [20, 29, 30] and mimicked attraction to the natural cotton headspace in the wind tunnel [30]. We selected five of these components to prepare an attractive synthetic mixture (Mix-5; amounts in Additional file 2): β-myrcene (95 %, Fluka), nonanal (95 %, Sigma-Aldrich), (*R*)-(+)-limonene (97 %, Sigma-Aldrich), (*E*)-β-ocimene (racemic, 90 %, Fluka), and (*Z*)-3-hexenyl acetate (98 %, Sigma-Aldrich). All components were diluted in ethanol and tested for attraction of either virgin males or mated females. (*Z*)-9-(*E*)-11-tetradecenyl acetate (*Z*9,*E*11-14:OAc, 10 pg.μL^−1^) and different concentrations of DMNT (0.001–1 ng.μL^−1^) were also diluted in ethanol and tested for attraction of virgin males only. DMNT (95 %) and *Z*9,*E*11-14:OAc (95 %) were gifts from Prof. Wittko Francke.

Cotton headspace collections and synthetic odour blends were delivered from the centre of the upwind end of the wind tunnel using a piezo-electric spraying device [68]. Cotton headspace collections were tested at 1,800 ng.h^−1^ of DMNT, the main compound in the collection (for chemical analysis of the headspace) (see [30]).

### GC-MS analyses

Volatile collections were analysed on a gas chromatograph-mass spectrometer (GC-MS; 6890 GC and 5975 MS, Agilent Technologies, Palo Alto, CA, USA). The GC was equipped with a fused silica capillary column (30 m × 0.25 mm, df = 0.25 μm) and DB-Wax (J&W Scientific, Folsom, CA, USA), and helium was used as the carrier gas at an average linear flow of 35 cm.s^−1^. The temperature was programmed from 30 °C (3 min hold) at 8 °C.min^−1^ to 225 °C (5 min hold). Identification of enantiomers of limonene was done on a fused silica capillary column (30 m × 0.25 mm) coated with HP-chiral 20B (df = 0.25 μm; Agilent), and the GC temperature was programmed from 30 °C (3 min hold) at 8 °C.min^−1^ to 225 °C (10 min hold). Compounds were identified by injection of authentic synthetic standards, retention times, Kovats indices, and library mass spectra (NIST, Agilent).

### Odour stimulation for SSR and Ca^2+^ imaging

In order to simulate the most attractive cotton blend (Mix-5) as vapour phase in a stimulus pipette, all compounds were carefully mixed to reproduce the ratio of odorants used in our wind tunnel assay. Differences in volatility of test compounds due to vapour pressure and van der Waals forces in paraffin oil were empirically corrected. β-myrcene, ocimene, (*R*)-(+)-limonene, (*Z*)-3-hexenyl acetate and nonanal were mixed together in paraffin oil at concentrations of 350, 280, 60, 120 and 330 ng.μL^−1^, respectively, and here onward called Mix-5 concentration 2. Its emission rate was assessed by applying 10 μL of the mixture on a piece of filter paper inside an Eppendorf tube closed with a 1-mL pipette tip. After 30 min resting at room temperature, 200 μL of the headspace was sampled using a gas-tight syringe (1.0 mL, Hamilton) and injected in the GC-MS for quantification. A 10x diluted solution of the synthetic cotton blend was prepared and here onward called Mix-5 concentration 1. DMNT was also diluted in paraffin oil in a separate vial at 0.01, 0.1 and 1 μg.μL^−1^.

GC-MS analyses of the headspace of Mix-5 (concentration 1) showed that this mixture provided a ratio of 1 (β-myrcene; 22 ± 2 μg.μL^−1^): 0.44 (ocimene; 10 ± 1 μg.μL^−1^): 0.15 ((*R*)-(+)-limonene; 3 ± 0.3 μg.μL^−1^): 0.74 ((*Z*)-3-hexenyl acetate; 16 ± 1 μg.μL^−1^): 0.96 (nonanal; 21 ± 2 μg.μL^−1^), which is similar to Mix-5 used in our wind tunnel assays (1:0.54:0.27:0.78:0.78, respectively; see Additional file 2). Addition of 10 μg DMNT to a second filter paper in the same stimulus pipette did not affect release rates of any stimuli in Mix-5 (see Additional file 2; d.dev = 0.645, df = 5, *P* = 0.596) and yielded a headspace concentration of DMNT of 81 ± 5 μg.μL^−1^ (see Additional file 2). Thus, these mixtures in paraffin oil were used in our following Ca^2+^ imaging and electrophysiology recordings.

The main pheromone component, *Z*9,*E*11-14:OAc, was mixed in hexane (Sigma-Aldrich) at concentrations of 1 and 10 μg.μL^−1^. However, measurements of the main pheromone component were not possible due to the low volatility of this type of compound [69].

We compared the activity of DMNT to that of suppressive compounds (*R*)-(−)-linalool (95 %, Firmenich) and (*S*)-(+)-linalool (95 %, Firmenich). Both enantiomers were diluted in paraffin oil at concentrations of 0.003, 0.03 and 0.3 μg.μL^−1^, as their volatility was approximately 3x higher than that of DMNT.

To prepare odour stimuli, two pieces of filter paper (10 × 15 mm) were placed inside glass Pasteur pipettes. One piece was loaded with 10 μL of either plant blends, whereas the next piece was loaded with 10 μL of either paraffin oil (control) or one of the DMNT dilutions. Pheromone stimuli were applied on a paper, and hexane was allowed to evaporate for 30 min in a fume hood. Pipettes were flushed with an air stream to remove all hexane prior to loading paraffin oil or DMNT solutions onto the second paper. All stimulus pipettes were then closed with a 1-mL pipette tip and left for 30 min prior to recordings. Either linalool enantiomer was prepared with *Z*9,*E*11-14:OAc in the same manner as described above for DMNT. Female moths were stimulated with plant odorants only. Each male was tested with either plant odorants or pheromone.

### Single sensillum recordings

A male moth was restrained in a plastic pipette tip with only the head protruding from the aperture, and a tungsten wire serving as a reference electrode was inserted into the abdomen. Single sensillum recordings (SSRs) were performed under a light microscope (Nikon FN-S2N) with 750x magnification, using tungsten electrodes (Clark Instruments Ltd). The recording electrode was attached to an AC/DC 10× gain probe (INR-02; Syntech), and its tip was inserted at the base of a pheromone-sensitive long trichoid sensillum using a micromanipulator (Märzhauser PM-10) until extracellular electrical contact with olfactory sensory neurons (OSNs) was established. The signal was amplified, digitized (IDAC-4 USB; Syntech) and visualized with AutoSpike 3.7 software (Syntech). A stream of charcoal filtered humidified air was continuously flushed over the antenna (1 L.min^−1^), through a glass tube (1.0 mm i.d.), which terminated 2.0 cm from the antenna. During stimulation, a 0.5-s air pulse (1.0 L min^−1^) controlled by a stimulus controller (CS-55; Syntech) was passed through the stimulus pipette, which was inserted into a hole in the glass tube. Compounds were tested with an inter-stimulus interval of at least 1 min. The response of OSNs was expressed as the number of spikes during the stimulation period after stimulus onset minus the number of spikes before stimulus onset.

### Calcium imaging

Moths were immobilized in pipette tips (1 mL) and dental wax for dissection. Cuticle, muscle fibres and trachea surrounding the brain were removed to fully access the ALs. The calcium-sensitive dye (CaGR-1-AM, Molecular Probes, Eugene, OR) was dissolved in 20 % Pluronic F-127 in dimethyl sulfoxide (Molecular Probes) and diluted in moth saline. The brain was covered with 50 μL of the dye solution and preparations were placed inside a box with a wet tissue for 1.5 h.

One AL was positioned under the microscope using a micromanipulator. Recordings were done using a TILL Photonics air-cooled imaging system (Gräfelfing, Germany) with a 12-bit slow-scan CCD camera. Sequences of 45 frames and a sampling rate of 5 Hz (150-ms exposure time at 470-nm excitation wavelength; Polychrome II) were recorded through an upright microscope (BX50WI, Olympus, Hamburg, Germany) with a 20x (NA 0.50; Olympus) water immersion objective. Fluorescence was detected with a dichroic filter (DCLP500 and LP515 emission filter).

An odour delivery glass tube (2.0 cm i.d.) was positioned approximately 1.0 cm distant from the antennae. A constant flow of clean humidified air was supplied through the tube at a rate of 1.0 L.min^−1^. Stimuli pipettes inserted inside the tube delivered odorants at a flow of 0.75 L.min^−1^ for 1 s using a stimulus controller (Syntech). A second empty pipette was placed next to the stimuli pipette and provided a continuous air flow (0.75 L.min^−1^) that was switched off during stimulation, keeping the total flow constant. Images were captured using 4 × 4 binning (160 × 120 pixels).

### Image processing

All image recordings were analysed with the neuroimage plugin [70] for the data analysis platform KNIME (KoNstanz Information MinEr). A signal processing approach, the convex cone algorithm [70], was employed to perform functional segmentation of the image plane into individual glomeruli and background in each animal. Glomeruli identification relied on functional and morphological data. Activation patterns of glomeruli to reference stimuli, that is, 10 μg α-humulene, DMNT, β-myrcene, ocimene, (*R*)-(+)-limonene, (*Z*)-3-hexenyl acetate and nonanal, constituted the main method used for recognition. Glomeruli were numbered according to a morphological atlas of *S. littoralis* male [31] and female ALs [29].

Kinetic data were exported to the statistical computing software R (version 3.0.3; [71]). Background fluorescence (average of frames 9–12) was subtracted and divided from all frames to yield the relative change in fluorescence (∆F/F). Bleaching was corrected by fitting appropriate negative exponential curves to each response curve [72]. Responses were normalized according to the response of an α-humulene-responding glomerulus to 10 μg of this compound (96 %, Sigma-Aldrich), a standard stimulus presented every four stimuli. Maximum response intensity was automatically calculated based on difference between the average value of frames 9–12 (before stimulus onset) and average of maximum intensity, one value before and two after.

### Statistical analysis

All statistical analyses were performed in R. Behavioural responses of male and female moths and concentrations of odorants in the vapour phase of synthetic plant odorants mixtures were analysed with binomial and gamma generalized linear models (glm), respectively. Spike frequencies of sensilla were analysed by Poisson glm. Maximum Ca^2+^ responses of glomeruli were analysed with linear mixed-effects models (lme) with individual insects as random effects using the lme4 package. Responses of single glomeruli to each concentration of either plant odorants or pheromone in combination with increasing doses of DMNT were analysed separately. *P* values of comparisons between treatment levels were calculated based on a *z*-distribution [73]. Significance of each variable in the model and difference in deviance (d.dev.) were assessed by comparing models with and without respective variables or interactions using a χ^2^ test.

## Additional files

Additional file 1:
**Test statistics of responses of individual glomeruli to (a) volatile cotton mimic and (b) single components.** Statistical tests: linear mixed-effect model. Myr: β-myrcene; Oci: (*E*)-β-ocimene; Lim: (*R*)-(+)-limonene; HexAc: (*Z*)-3-hexenyl acetate; Non: nonanal. (XLSX 11 kb) Additional file 2:
**Quantification of volatile compounds used in wind tunnel assays and in the headspace of stimulus pipettes.** Values are mean ± SE. No significant difference was found between Mix-5 and Mix-5:DMNT, and DMNT alone and DMNT within Mix-5:DMNT (d.dev = 0.645, df = 5, *P* = 0.596, n = 15). Myr: β-myrcene; Oci: (*E*)-β-ocimene; Lim: (*R*)-(+)-limonene; HexAc: (*Z*)-3-hexenyl acetate; Non: nonanal. (XLSX 54 kb)

## Abbreviations

AL: antennal lobe
d.dev.: difference in deviance
DMNT: (*E*)-4,8-dimethyl-1,3,7-nonatriene
GC-MS: gas chromatograph-mass spectrometer
HIPV: herbivore-induced plant volatile
OSN: olfactory sensory neuron
SSR: single sensillum recording
VOC: volatile organic compound
*Z*9*E*11–14OAc: (*Z*)-9-(*E*)-11-tetradecenyl acetate

## References

[1] Janz N. Evolutionary ecology of oviposition strategies. In: Hilker M, Meiners T, editors. Chemoecology of insect eggs and egg deposition*.* Oxford, UK: Blackwell Publishing Ltd.; 2002. p. 349–76.

[2] Davis JM, Stamps JA (2004). The effect of natal experience on habitat preferences. Trends Ecol Evol.

[3] Alcock J (2009). The control of behavior: neural mechanisms. Animal behavior: an evolutionary approach.

[4] Bruce TJA, Wadhams LJ, Woodcock CM (2005). Insect host location: a volatile situation. Trends Plant Sci.

[5] Dicke M, Vanbeek TA, Posthumus MA, Bendom N, Vanbokhoven H, Degroot AE (1990). Isolation and identification of volatile kairomone that affects acarine predator–prey interactions - involvement of host plant in its production. J Chem Ecol.

[6] Paré PW, Tumlinson JH (1999). Plant volatiles as a defense against insect herbivores. Plant Physiol.

[7] Turlings TCJ, Tumlinson JH, Lewis WJ (1990). Exploitation of herbivore-induced plant odors by host-seeking parasitic wasps. Science.

[8] Turlings TC, Loughrin JH, McCall PJ, Röse US, Lewis WJ, Tumlinson JH (1995). How caterpillar-damaged plants protect themselves by attracting parasitic wasps. Proc Natl Acad Sci U S A.

[9] Tamiru A, Bruce TJA, Woodcock CM, Caulfield JC, Midega CAO, Ogol CKPO (2011). Maize landraces recruit egg and larval parasitoids in response to egg deposition by a herbivore. Ecol Lett.

[10] Tollrian R, Harvell CD, Tollrian R, Harvell CD (1998). The evolution of inducible defences: current ideas. The ecology and evolution of inducible defences.

[11] Kessler A, Baldwin IT (2001). Defensive function of herbivore-induced plant volatile emissions in nature. Science.

[12] de Moraes CM, Mescher MC, Tumlinson JH (2001). Caterpillar-induced nocturnal plant volatiles repel conspecific females. Nature.

[13] Dicke M (1986). Volatile spider-mite pheromone and host-plant kairomone, involved in spaced-out gregariousness in the spider mite *Tetranychus urticae*. Physiol Entomol.

[14] Mack L, Gros P, Burkhardt J, Seifert K (2013). Elicitors of tansy volatiles from cotton leafworm larval oral secretion. Phytochemistry.

[15] Dicke M, van Loon JJA (2000). Multitrophic effects of herbivore-induced plant volatiles in an evolutionary context. Entomol Exp Appl.

[16] Arimura G, Ozawa R, Horiuchi J, Nishioka T, Takabayashi J (2001). Plant-plant interactions mediated by volatiles emitted from plants infested by spider mites. Biochem Syst Ecol.

[17] Engelberth J, Seidl-Adams I, Schultz JC, Tumlinson JH (2007). Insect elicitors and exposure to green leafy volatiles differentially upregulate major octadecanoids and transcripts of 12-oxo phytodienoic acid reductases in *Zea mays*. Mol Plant Microbe Interact.

[18] Mahanil S, Attajarusit J, Stout MJ, Thipyapong P (2008). Overexpression of tomato polyphenol oxidase increases resistance to common cutworm. Plant Sci.

[19] Dicke M (2009). Behavioural and community ecology of plants that cry for help. Plant Cell Environ.

[20] Zakir A, Bengtsson M, Sadek MM, Hansson BS, Witzgall P, Anderson P (2013). Specific response to herbivore-induced de novo synthesized plant volatiles provides reliable information for host plant selection in a moth. J Exp Biol.

[21] Pickett JA, Woodcock CM, Midega CAO, Khan ZR (2014). Push-pull farming systems. Curr Opin Biotechnol.

[22] Loughrin JH, Manukian A, Heath RR, Turlings TCJ, Tumlinson JH (1994). Diurnal cycle of emission of induced volatile terpenoids herbivore-injured cotton plants. Proc Natl Acad Sci U S A.

[23] Röse USR, Tumlinson JH (2005). Systemic induction of volatile release in cotton: how specific is the signal to herbivory?. Planta.

[24] Paré PW, Tumlinson JH (1997). De novo biosynthesis of volatiles induced by insect herbivory in cotton plants. Plant Physiol.

[25] Röse USR, Manukian A, Heath RR, Tumlinson JH (1996). Volatile semiochemicals released from undamaged cotton leaves (a systemic response of living plants to caterpillar damage). Plant Physiol.

[26] Röse USR, Tumlinson JH (2004). Volatiles released from cotton plants in response to *Helicoverpa zea* feeding damage on cotton flower buds. Planta.

[27] McCall P, Turlings TJ, Loughrin J, Proveaux A, Tumlinson J (1994). Herbivore-induced volatile emissions from cotton (*Gossypium hirsutum* L.) seedlings. J Chem Ecol.

[28] Hegde M, Oliveira J, da Costa J, Bleicher E, Santana AG, Bruce TA (2011). Identification of semiochemicals released by cotton, *Gossypium hirsutum*, upon infestation by the cotton aphid, *Aphis gossypii*. J Chem Ecol.

[29] Saveer AM, Kromann SH, Birgersson G, Bengtsson M, Lindblom T, Balkenius A (2012). Floral to green: mating switches moth olfactory coding and preference. Proc R Soc B Biol Sci.

[30] Borrero-Echeverry F, Becher PG, Birgersson GÅO, Bengtsson M, Witzgall P, Saveer AM. Flight attraction of *Spodoptera littoralis* (Lepidoptera, Noctuidae) to cotton headspace and synthetic volatile blends. Front Ecol Evol. 2015;3.

[31] Couton L, Minoli S, Kieu K, Anton S, Rospars JP (2009). Constancy and variability of identified glomeruli in antennal lobes: computational approach in *Spodoptera littoralis*. Cell Tissue Res.

[32] Pregitzer P, Schubert M, Breer H, Hansson BS, Sachse S, Krieger J (2012). Plant odorants interfere with detection of sex pheromone signals by male *Heliothis virescens*. Front Cell Neurosci.

[33] Ignell R, Root CM, Birse RT, Wang JW, Nassel DR, Winther AME (2009). Presynaptic peptidergic modulation of olfactory receptor neurons in *Drosophila*. Proc Natl Acad Sci U S A.

[34] Olsen SR, Wilson RI (2008). Lateral presynaptic inhibition mediates gain control in an olfactory circuit. Nature.

[35] Reisenman CE, Riffell JA, Duffy K, Pesque A, Mikles D, Goodwin B (2013). Species-specific effects of herbivory on the oviposition behavior of the moth *Manduca sexta*. J Chem Ecol.

[36] Hillier NK, Vickers NJ (2011). Mixture interactions in moth olfactory physiology: examining the effects of odorant mixture, concentration, distal stimulation, and antennal nerve transection on sensillar responses. Chem Senses.

[37] Galizia CG, Rossler W (2010). Parallel olfactory systems in insects: anatomy and function. Annu Rev Entomol.

[38] Renou M, Mucignat-Caretta C (2014). Pheromones and general odor perception in insects. Neurobiology of chemical communication.

[39] Zakir A, Sadek MM, Bengtsson M, Hansson BS, Witzgall P, Anderson P (2013). Herbivore-induced plant volatiles provide associational resistance against an ovipositing herbivore. J Ecol.

[40] Paré PW, Tumlinson JH (1997). Induced synthesis of plant volatiles. Nature.

[41] Donath J, Boland W (1994). Biosynthesis of acyclic homoterpenes in higher plants parallels steroid hormone metabolism. J Plant Physiol.

[42] Visser JH, Avé DA (1978). General green leaf volatiles in the olfactory orientation of the Colorado beetle, *Leptinotarsa decemlineata*. Entomol Exp Appl.

[43] Stensmyr MC, Dweck HKM, Farhan A, Ibba I, Strutz A, Mukunda L (2012). A conserved dedicated olfactory circuit for detecting harmful microbes in *Drosophila*. Cell.

[44] van der Pers JNC, Thomas G, Denotter CJ (1980). Interactions between plant odors and pheromone reception in small ermine moths (Lepidoptera: Yponomeutidae). Chem Senses.

[45] Party V, Hanot C, Said I, Rochat D, Renou M (2009). Plant terpenes affect intensity and temporal parameters of pheromone detection in a moth. Chem Senses.

[46] Chaffiol A, Kropf J, Barrozo RB, Gadenne C, Rospars JP, Anton S (2012). Plant odour stimuli reshape pheromonal representation in neurons of the antennal lobe macroglomerular complex of a male moth. J Exp Biol.

[47] Lei H, Vickers N (2008). Central processing of natural odor mixtures in insects. J Chem Ecol.

[48] Steullet P, Derby CD (1997). Coding of blend ratios of binary mixtures by olfactory neurons in the Florida spiny lobster, *Panulirus argus*. J Comp Physiol A Sens Neural Behav Physiol.

[49] Cromarty SI, Derby CD (1998). Inhibitory receptor binding events among the components of complex mixtures contribute to mixture suppression in responses of olfactory receptor neurons of spiny lobsters. J Comp Physiol A.

[50] Oka Y, Omura M, Kataoka H, Touhara K (2004). Olfactory receptor antagonism between odorants. EMBO J.

[51] Wetzel CH, Brunert D, Hatt H (2005). Cellular mechanisms of olfactory signal transduction. Chem Senses.

[52] Schneider D, Lacher V, Kaissling K-E (1964). Die Reaktionsweise und das Reaktionsspektrum von Riechzellen bei *Antheraea pernyi* (Lepidoptera, Saturniidae). Z Vergl Physiol.

[53] Riffell JA, Lei H, Christensen TA, Hildebrand JG (2009). Characterization and coding of behaviorally significant odor mixtures. Curr Biol.

[54] Riffell JA, Lei H, Hildebrand JG (2009). Neural correlates of behavior in the moth *Manduca sexta* in response to complex odors. Proc Natl Acad Sci U S A.

[55] Rospars J-P, Lansky P, Chaput M, Duchamp-Viret P (2008). Competitive and noncompetitive odorant interactions in the early neural coding of odorant mixtures. J Neurosci.

[56] Münch D, Schmeichel B, Silbering AF, Galizia CG (2013). Weaker ligands can dominate an odor blend due to syntopic interactions. Chem Senses.

[57] Root CM, Masuyama K, Green DS, Enell LE, Nassel DR, Lee CH (2008). A presynaptic gain control mechanism fine-tunes olfactory behavior. Neuron.

[58] McGann JP (2013). Presynaptic inhibition of olfactory sensory neurons: new mechanisms and potential functions. Chem Senses.

[59] Su CY, Menuz K, Reisert J, Carlson JR (2012). Non-synaptic inhibition between grouped neurons in an olfactory circuit. Nature.

[60] Binyameen M, Anderson P, Ignell R, Seada MA, Hansson BS, Schlyter F (2012). Spatial organization of antennal olfactory sensory neurons in the female *Spodoptera littoralis* moth: differences in sensitivity and temporal characteristics. Chem Senses.

[61] Jones PL, Pask GM, Rinker DC, Zwiebel LJ (2011). Functional agonism of insect odorant receptor ion channels. Proc Natl Acad Sci U S A.

[62] Dekker T, Geier M, Cardé RT (2005). Carbon dioxide instantly sensitizes female yellow fever mosquitoes to human skin odours. J Exp Biol.

[63] Turner SL, Ray A (2009). Modification of CO_2_ avoidance behaviour in *Drosophila* by inhibitory odorants. Nature.

[64] Turner SL, Li N, Guda T, Githure J, Cardé RT, Ray A (2011). Ultra-prolonged activation of CO_2_-sensing neurons disorients mosquitoes. Nature.

[65] Hassanali A, Herren H, Khan ZR, Pickett JA, Woodcock CM (2008). Integrated pest management: the push-pull approach for controlling insect pests and weeds of cereals, and its potential for other agricultural systems including animal husbandry. Phil Trans R Soc B.

[66] Khan ZR, Pickett JA, van den Berg J, Wadhams LJ, Woodcock CM (2000). Exploiting chemical ecology and species diversity: stem borer and striga control for maize and sorghum in Africa. Pest Manag Sci.

[67] Bisch-Knaden S, Carlsson MA, Sugimoto Y, Schubert M, Mißbach C, Sachse S (2012). Olfactory coding in five moth species from two families. J Exp Biol.

[68] El-Sayed A, Godde J, Arn H (1999). Sprayer for quantitative application of odor stimuli. Environ Entomol.

[69] Bengtsson M, Liljefors T, Hansson BS, Lofstedt C, Copaja SV (1990). Structure-activity relationships for chain-shortened analogs of (*Z*)-5-decenyl acetate, a pheromone component of the turnip moth, *Agrotis segetum*. J Chem Ecol.

[70] Strauch M, Rein J, Lutz C, Galizia CG (2013). Signal extraction from movies of honeybee brain activity: the ImageBee plugin for KNIME. BMC Bioinformatics.

[71] Team TRC (2013). R: A Language and Environment for Statistical Computing.

[72] Stetter M, Greve H, Galizia CG, Obermayer K (2001). Analysis of calcium imaging signals from the honeybee brain by nonlinear models. Neuroimage.

[73] Zuur AF, Hilbe JM, Ieno EN (2013). Introduction to mixed effects model. A beginner’s guide to GLM and GLMM with R: a frequentist and Bayesian perspective for ecologists.

